# Transplantation of fecal microbiota from patients with inflammatory bowel disease and depression alters immune response and behavior in recipient mice

**DOI:** 10.1038/s41598-021-00088-x

**Published:** 2021-10-14

**Authors:** Hyo-Min Jang, Jeon-Kyung Kim, Min-Kyung Joo, Yoon-Jung Shin, Chang Kyun Lee, Hyo-Jong Kim, Dong-Hyun Kim

**Affiliations:** 1 Neurobiota Research Center, College of Pharmacy, Kyung Hee University, 26, Kyungheedae-ro, Dongdaemun-gu, Seoul, 02447 Korea; 2 Department of Internal Medicine, Kyung Hee University School of Medicine, Seoul, 02447 Korea

**Keywords:** Immunology, Microbiology, Neuroscience

## Abstract

Gut dysbiosis is closely associated with the occurrence of inflammatory bowel disease (IBD) and psychiatric disorder. Here, to understand the difference of gut microbiota composition and physiological effect between IBD patients with (IBD/D^+^) or without depression (IBD/D^−^), we analyzed the fecal microbiota composition of patients with IBD with (/D^+^) or without depression (/D^−^) and healthy volunteers (HVs) and examined the effects of these fecal microbiota transplantations (FMTs) on the occurrence of systemic inflammation and anxiety/depression in mice. FMTs from patients with IBD/D^+^ or IBD/D^−^ caused IBD-like colitis in the transplanted mice: they increased the myeloperoxidase activity, IL-1β and IL-6 expression, and NF-κB^+^/CD11c^+^ cell population in the colon. Transplantation of the IBD/D^+^ patient feces (IBD/D^+^-F) caused IBD-like colitis more strongly than that of IBD/D^−^-F. FMTs from patients with IBD/D^+^ also caused anxiety-/depression-like behaviors, increased the NF-κB^+^/Iba1^+^ and lipopolysaccharide (LPS)^+^/Iba1^+^ cell populations, and decreased the BDNF^+^/NeuN^+^ cell population in the hippocampus. They increased LPS levels in the blood. FMTs from patients with IBD/D^−^ caused anxiety-like, but not depression-like, behaviors. α-/β-diversities and composition of gut microbiota in IBD-F were different from those of HV feces (HV-F). The *Enterobacteriaceae* and *Enterococcaceae* populations and LPS levels were higher in the IBD-F than in the HV-F. The *Enterococcaceae* population was higher in IBD/D^+^-F vs. IBD/D^−^-F. However, the transplantation of HV-F into mice previously transplanted with IBD/D^+^-F significantly reduced depression-like behaviors, NF-κB^+^/Iba1^+^ and LPS^+^/Iba1^+^ cell populations in the hippocampus, LPS levels in the feces and blood, and IL-1β expression in the colon. These findings suggest that the outbreak of depression/anxiety may be dependent on the systemic inflammation with a leaky gut through the gut dysbiosis-attributable overproduction of bacterial LPS and suppression of tight junction protein expression in patients with IBD.

## Introduction

Inflammatory bowel disease (IBD), including ulcerative colitis (UC) and Crohn’s disease (CD), is a chronic and relapsing–remitting inflammatory disorder of the gastrointestinal tract^1^. The pathogenesis of IBD is generally known to be the dysfunction of the mucosal immune system toward commensal gut microbiota with genetic and environmental factors^2,3^. Interestingly, the prevalence of psychiatric disorders such as anxiety and depression is significantly higher in patients with IBD than in healthy personnel^4,5^. Evidence in support of the close connection between IBD and psychiatric disorders stems primarily from animal and human studies^5,6^. Exposure to stressors such as social defeat and immobilization stress (IS) induces the release of interleukin (IL)-6 and tumor necrosis factor (TNF)-α in the colon mucosa of patients with UC or CD^7–9^. In the Manitoba IBD Cohort Study, 80% of patients with both IBD and mood disorder are diagnosed with mood disorder before the occurrence of IBD^10^. Exposure of mice to stressors, such as IS and pathogen infections, stimulates the release of adrenal hormones, such as cortisol, in the adrenal gland and immune cytokines, such as IL-1β and IL-6, in the immune cells through the hypothalamus − pituitary − adrenal (HPA) axis activation, leading to gut inflammation and dysbiosis accompanied by psychiatric disorders^8,11–13^. Anti-inflammatory drugs alleviate colitis as well as psychiatric disorders^7^. Anti-depressant drugs improve depression as well as colitis^14^. These findings suggest that the brain is closely connected with the gut via the HPA and gut − brain axes.

The gut microbiome of humans and animals is composed of bacteria, archaea, fungi, viruses, and multicellular parasites^15,16^. Of these microbes, commensal opportunistic pathogens, including gram-negative microbes, secrete toxic byproducts, including lipopolysaccharide (LPS)^17,18^. Of these pathogens, *Klebsiella oxytoca* and *Escherichia coli* are overgrown in the gastrointestinal tract by exposure to stressors, such as antibiotics and IS^11,12^. They strongly secrete endotoxins, which dysregulate gut immune homeostasis, induce inflammatory cytokines, and suppress tight junction protein expression, leading to the outbreak of gut inflammation with a leaky gut, as previously reported in patients with IBD^8,11,19,20^. Furthermore, exposure to LPS causes a leaky gut with gut inflammation^11,20,21^. The leaky gut accelerates the absorption of bacterial byproducts, such as LPS, into the blood and alters the gut microbiota composition, which is termed dysbiosis^12,19,20,22^. In addition, the gut microbial α-diversities of patients with IBD are lower compared with those of healthy individuals^23,24^. Their β-diversities are also significantly different^19,23^. The Proteobacteria and Firmicutes populations were significantly higher in patients with IBD than in healthy individuals^25^. Gut inflammation increases the Proteobacteria population in mice^11–13^. The fecal microbiota transplantation (FMT) from IBD patients causes the IBD-like colitis in germ-free mice^26,27^. FMT from mice with colitis occurs colitis as well as depression in conventional mice^11^. FMT from healthy individuals alleviates IBD in clinic studies^27,28^. These findings suggest that the microbiota are closely connected with the occurrence of IBD and neuropsychiatric disorders, such as depression, through the microbiota − gut − brain (MGB) axis. Nevertheless, the difference of gut microbiota composition between IBD patients with (IBD/D^+^) or without depression (IBD/D^−^) remains unclear. Nevertheless, many studies are focused on the association between the gut microbiota and pathogenesis in IBD^3,16,19^.

In the present study, we transplanted the fecal microbiota of patients with IBD/D^+^ or IBD/ /D^−^ into specific-pathogen-free mice or healthy human volunteers (HVs) into mice transplanted with the feces of patients with IBD/D^+^ and examined their effects regarding the occurrence of colitis, anxiety/depression, and gut microbiota composition.

## Results

### Fecal microbiota transplantations from patients with IBD caused colitis as well as anxiety/depression in the transplanted mice

**Figure 1:**
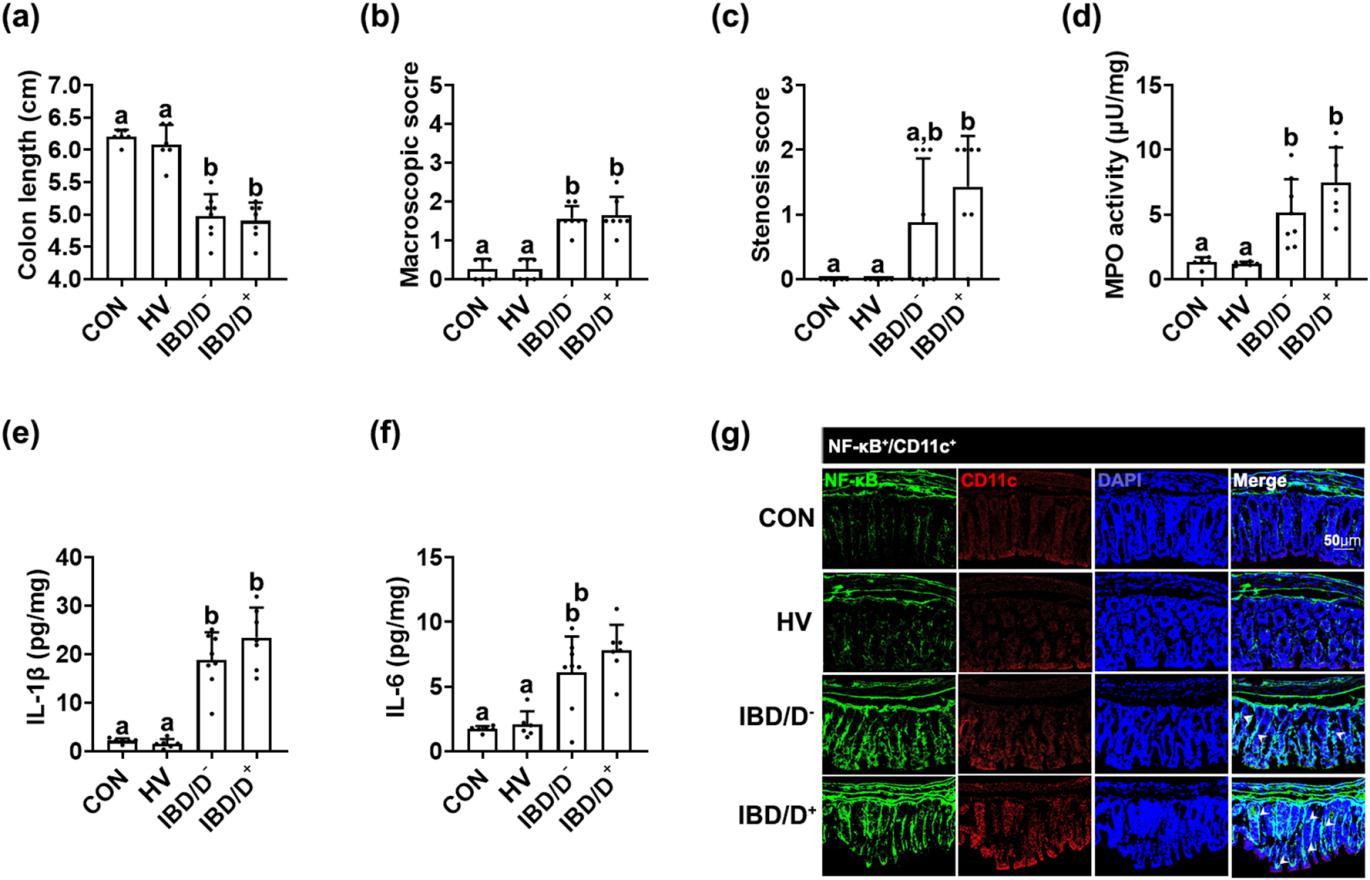
Fecal microbiota transplantation from patients with IBD/D^+^ or IBD/D^−^ and healthy volunteers (HVs) caused colitis in the transplanted mice. Effects on the colon length (**a**), macroscopic score (**b**), stenosis score (**c**), myeloperoxidase activity (**d**), IL-1β (**e**) and IL-6 expression (**f**), and NF-κB^+^/Iba1^+^ cell population (**g**) in the colon. Each HV-F (n = 6), IBD/D^−^-F (n = 8), or IBD/D^+^-F (n = 7) was orally transplanted in three mice once a day for 5 days. Control mice were treated with vehicle (saline) instead of fecal suspension. Data values were indicated as mean ± SD (NC n = 6; HV-F n = 6; IBD/D^−^ n = 8; IBD/D^+^ n = 7: each n value is the average obtained from 3 mice). Means with same letters are not significantly different (*p* < 0.05). All data were analyzed using ANOVA with Tukey’s multiple comparisons test.

To understand the role of gut microbiota in the occurrence of IBD and anxiety/depression, we transplanted the fecal microbiota of HVs or patients with IBD/D^+^ (score ≥ 11 on HADS-D) or IBD/D^−^ (score ≤ 6 on HADS-D) into specific pathogen-free mice and examined their effects regarding the occurrence of colitis and anxiety/depression. The FMTs from patients with IBD/D^+^ or IBD/D^−^ all caused colitis in mice: they significantly induced colon shortening; upregulated myeloperoxidase activity, IL-1β and IL-6 expression and increased the NF-κB^+^/CD11c^+^ cell population in the colon (Fig. 1a–g). In contrast, the FMT from HVs did not cause colitis. In particular, the stenosis was a significant occurrence in mice with FMTs from IBD/D^+^ patients vs. mice with FMTs from IBD/D^−^ patients. After segregation of the IBD population into patients with patients with UC or CD, the FMTs of patients with UC or CD all caused colitis in mice, regardless of the presence/absence of depression: they increased colon shortening; upregulated myeloperoxidase activity, IL-1β and IL-6 expression; increased the NF-κB^+^/CD11c^+^ cell population in the colon (Supplementary Figure S1). Although the occurrence of IBD-like colitis between FMTs from UC and CD patients was not significantly different, the stenosis occurrence was higher in mice with FMTs from patients with CD than in one with UC, but was not different between ones transplanted with the feces of HVs and UC patients.

**Figure 2:**
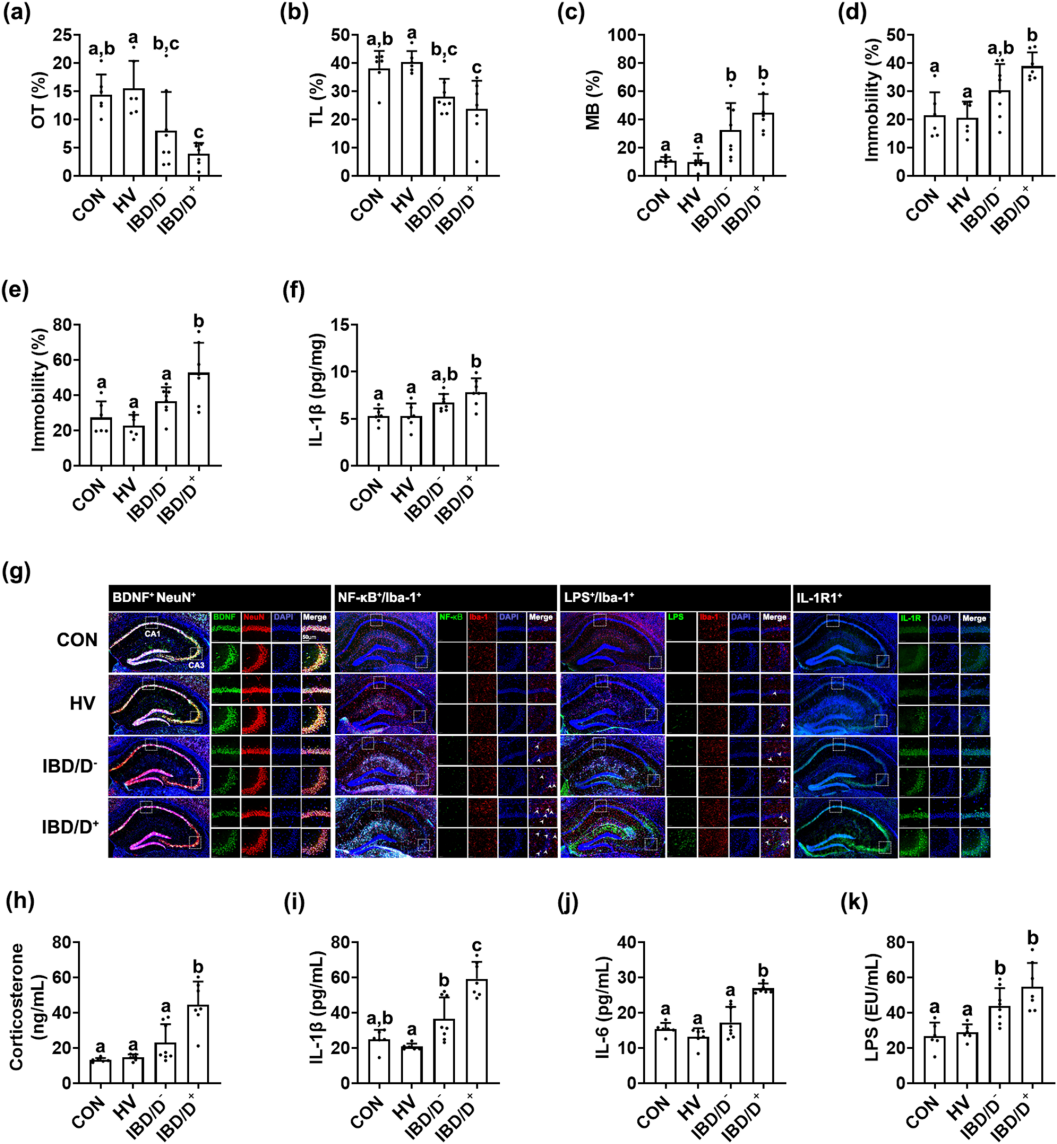
Fecal microbiota transplantation from patients with IBD/D^−^ or IBD/D^+^ and healthy volunteers (HVs) caused anxiety/depression in the transplanted mice. Effects on the occurrence of anxiety/depression in the EPM (**a**), LDT (**b**), MB tasks (**c**), TST (**d**), and FST (**e**). Effects on the IL-1β expression (**f**), BDNF^+^/NeuN^+^, NF-κB^+^/Iba1^+^, LPS^+^/Iba1^+^ and IL-1R^+^ cell populations in the hippocampus (**g**). Effects on the corticosterone (**h**), IL-1β (**i**), IL-6 (**j**), and LPS levels (**k**). Each HV-F (n = 6), IBD/D^−^-F (n = 8), or IBD/D^+^-F (n = 7) was orally transplanted in three mice once a day for 5 days. Control mice were treated with vehicle (saline) instead of fecal suspension. Data values were indicated as mean ± SD (NC n = 6; HV-F n = 6; IBD/D^−^ n = 8; IBD/D^+^ n = 7: each n value is the average obtained from 3 mice). Means with same letters are not significantly different (*p* < 0.05). All data were analyzed using ANOVA with Tukey’s multiple comparisons test.

The FMTs from patients with IBD/D^+^ or IBD/D^−^ all also significantly increased anxiety-like behaviors in the transplanted mice in the EPM, LDT, and MB tasks (Fig. 2a–e). Moreover, depression-like behaviors in the TST and FST were significantly increased by the FMTs from patients with IBD/D^+^, but not those from patients with IBD/D^−^. The FMTs from patients with IBD also increased NF-κB^+^/Iba1^+^, and LPS^+^/Iba1^+^, and IL-1R^+^ cell populations, as well as IL-1β expression in the hippocampus, while the BDNF^+^/NeuN^+^ cell population reduced (Fig. 2f,g). They also increased corticosterone, IL-1β, IL-6, and LPS levels in the blood (Fig. 2h–k). After the segregation of the IBD population into patients with UC and CD, the FMTs from patients with UC and CD caused depression-like behaviors in mice (Supplementary Figure S2). The neuroinflammatory markers IL-1β and IL-6 and the NF-κB^+^/Iba1^+^ cell population were induced in the hippocampus of mice with FMT from patients with UC and CD. However, the FMT from HVs did not significantly affect neuroinflammation markers.

### Fecal microbiota composition in patients with IBD/D^+^ and IBD/D^−^

**Figure 3:**
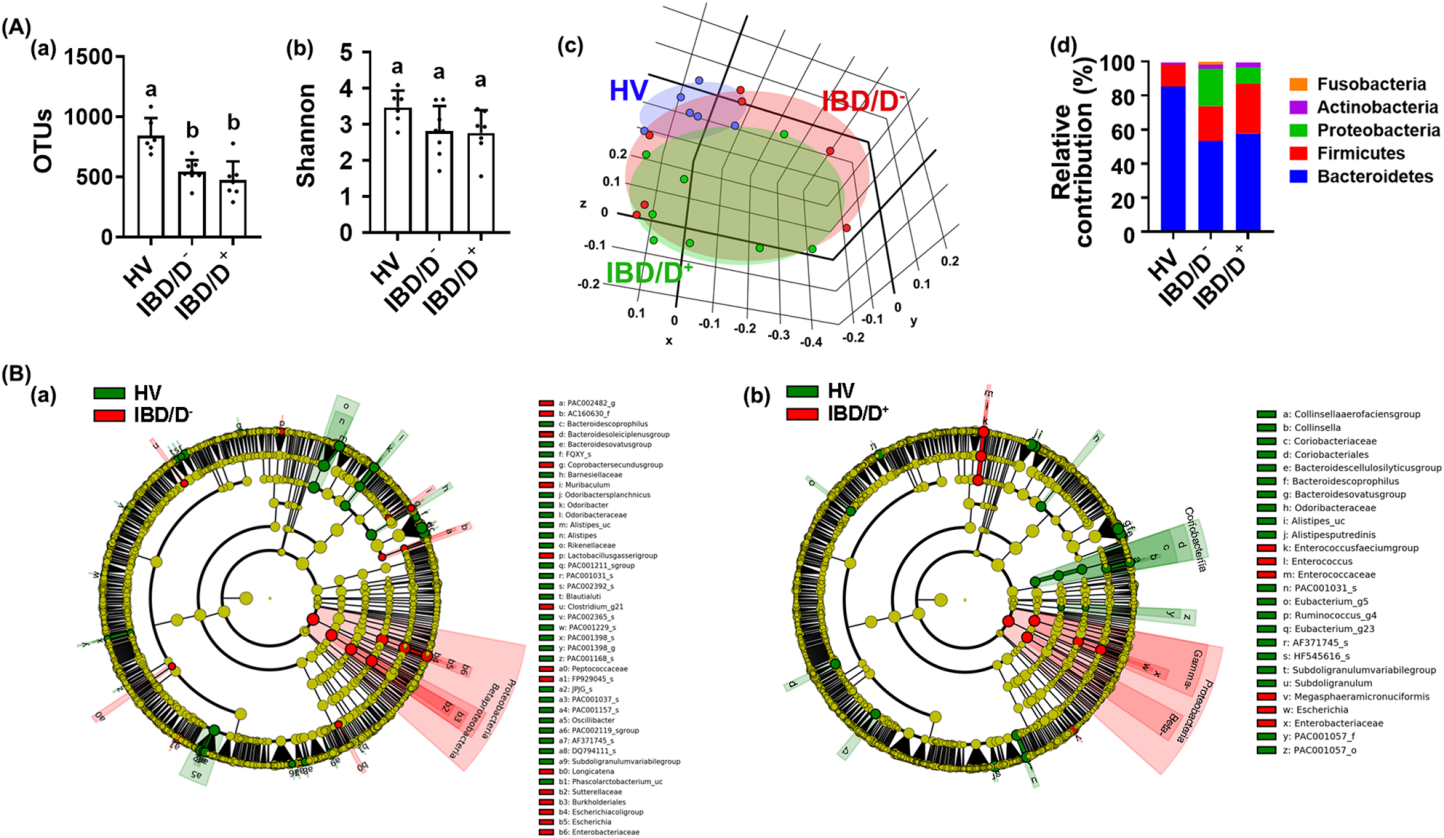
The fecal microbiota composition of patients with IBD/D^−^ or IBD/D^+^ and healthy volunteers (HVs). (**A**) Effects on OTU richness (a), Shannon’s index (b), principal coordinate analysis (PCoA) plot based on Jensen-Shannon analysis (c), and phylum level (d). (**B**) Effects on gut microbiota composition indicated by Cladogram between the feces of HVs and IBD/D^−^ patients (a) and between the feces of HVs and IBD/D^+^ patients (b). The gut microbiota composition was analyzed by using using Illumina iSeq 100. Data values were indicated as mean ± SD (HV n = 6; IBD/D^−^ n = 8; IBD/D^+^ n = 7). Means with same letters are not significantly different (*p* < 0.05). (A)(a, b) and (C)(b, c, d), Kruskal–Wallis test (nonparametric test); (C)(a), One-way ANOVA Bonferroni’s multiple comparisons test (parametric test); (C)(e), Mann Whitney test (nonparametric test).

The FMTs from patients with IBD significantly caused colitis and anxiety/depression in transplanted mice compared with those from HVs. Therefore, to understand the role of gut microbiota in the occurrence of depression and colitis, we investigated the fecal microbiota composition of the HV-F, IBD/D^+^-F, and IBD/D^−^-F using pyrosequencing (Fig. 3, Supplementary Figures S3 and S4). The α-diversity estimated operational taxonomic unit (OTU) richness, but not Shannon’s diversity index, was lower in the IBD-F than in the HV-F (Fig. 3A,B). Moreover, the OTU richness was lower in the IBD/D^+^-F than in the IBD/D^−^-F. The β-diversity and bacterial community were significantly different between IBD/D^+^-F and IBD/D^−^-F. At the phylum level, the Proteobacteria population was higher in the IBD-F compared with HV-F, while the Bacteroidetes population was lower in the IBD-F. At the family level, the populations of *Enterobacteriaceae* and *Enterococcaceae* were higher in the IBD-F compared with HV-F (Fig. 3C). The *Enterococcaceae* and *Lactobacillaceae* populations were higher in the IBD/D^+^-F than in the IBD/D^−^-F. In particular, the fecal LPS level was significantly higher in the IBD-F than in the HV-F (Supplementary Figure S5). It was weakly, but not significantly, higher in the IBD/D^+^-F than in the IBD/D^−^-F.

**Figure 4:**
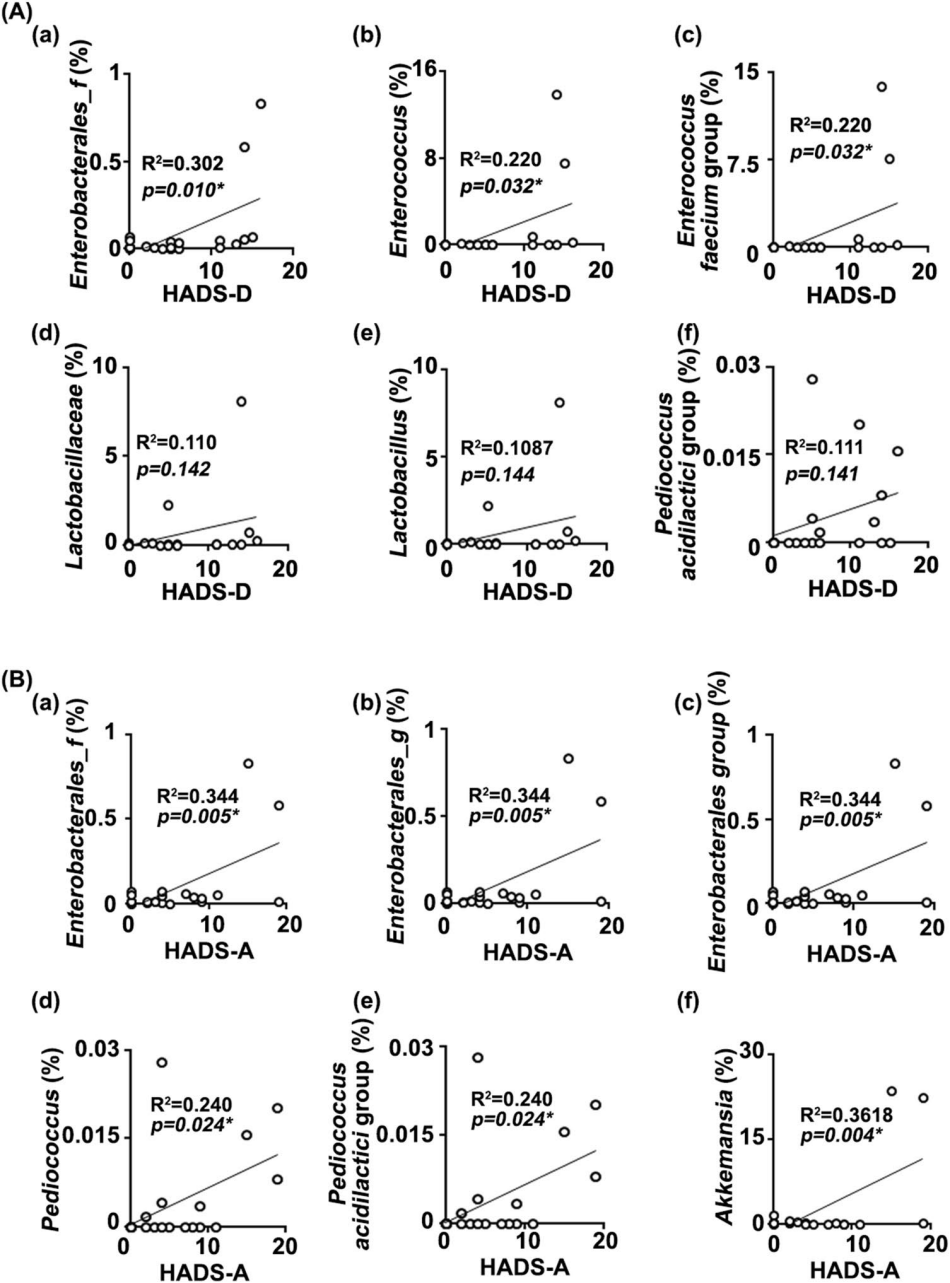
The relationship between HADS and gut microbiota composition. (**A**) The relationship between HADS-D and gut microbiota composition: (a) Enterobacterales_f, (b) *Enterococcus*, (c) *Enterococcus faecium* group, (d) *Lactobacillaceae,* (e) Lactobacillus, and (f) *Pediococcus acidilactici* group. (**B**) The relationship between HADS-A and gut microbiota composition: (a) Enterobacterales_f, (b) Enterobacterales_g, (c) Enerobacterales group, (d) Pediococcus, (e) *Pediococcus acidilactici* group, and (f) Akkemansia. HV n = 6; IBD/D^−^ n = 8; IBD/D^+^ n = 7.

Next, to understand what kinds of gut microbiota are associated with the occurrence of anxiety or depression, we analyzed the correlation between the gut microbiota composition and depression index in patients with IBD (hospital anxiety and depression scale: HADS-A and HADS-D) (Fig. 4). The Enterobacterales_f (R^2^ = 0.302, *p* = 0.010), *Enterococcus* (R^2^ = 0.220, *p* = 0.032), and *Lactobacillaceae* (R^2^ = 0.110, *p* = 0.142) populations were positively correlated with the depression index in patients with IBD (HADS-D) (Fig. 3A). The Enterobacterales_f (R^2^ = 0.344, *p* = 0.005), Enterobacterales_g (R^2^ = 0.344, *p* = 0.005), Enterobacterales group (R^2^ = 0.344, *p* = 0.005), and *Bacteroides uniformis* (R^2^ = 0.346, *p* = 0.005) populations were positively correlated with the anxiety index in patients with IBD (HADS-A) (Fig. 3B). In addition, even if two IBD/D + patients thought to be outliers were excluded, the correlation between the composition of gut bacteria, except *Enterococcus faecium* group, and depression index was not different to those included them (Supplementary Figure S6).

### Fecal microbiota composition in mice transplanted with IBD/D^+^-F or IBD/D^−^-F

**Figure 5:**
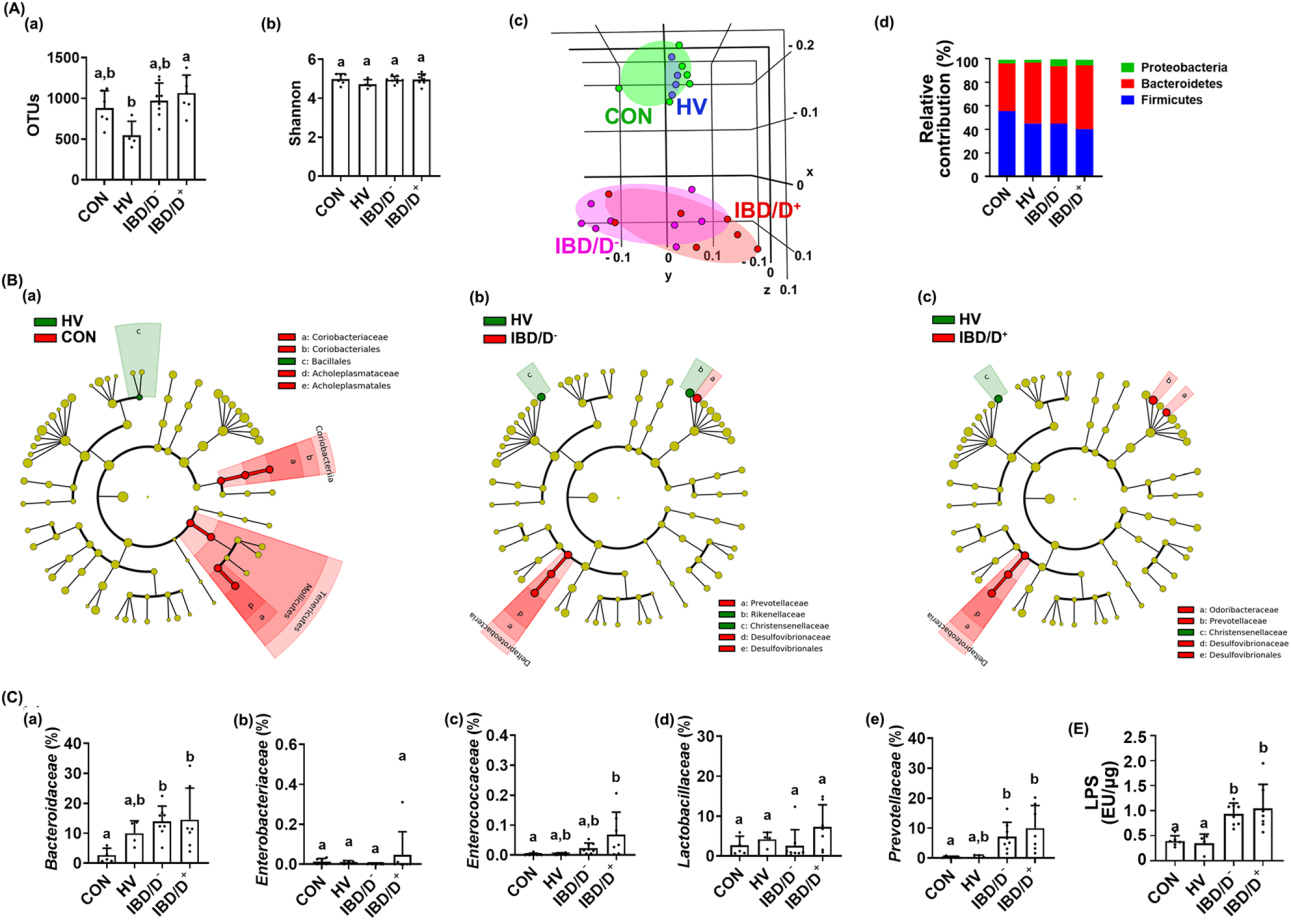
The fecal microbiota composition of mice orally transplanted with patients with IBD/D^−^ or IBD/D^+^ and healthy volunteers (HVs). (**A**) Effects on OTU richness (a), Shannon’s index (b), principal coordinate analysis (PCoA) plot based on Jensen-Shannon analysis (c), and phylum level (d). (**B**) Effects on gut microbiota composition indicated by Cladogram between the feces of normal control mice (CON) and mice transplanted with HV (a), between the feces of HVs and IBD/D^−^ patients (b) and between the feces of HVs and IBD/D^+^ patients (c). (**C**) Effects on the levels of families *Bacteroidaceae* (a), *Enterobacteriaceae* (b), *Enterococcaceae* (c), *Lactobacillaceae* (d), and *Prevotellaceae* (e). The gut microbiota composition was analyzed by using using Illumina iSeq 100. (D) Effects on the fecal LPS levels. Data values were indicated as mean ± SD (NC n = 6; HV-F n = 4; IBD/D^−^ n = 8; IBD/D^+^ n = 7: each n value is the average obtained from 3 mice). Means with same letters are not significantly different (*p* < 0.05). (A)(a,b), (C)(a,b,d,e), and (D), Kruskal–Wallis test (nonparametric test); (B)(c), ANOVA with Tukey’s multiple comparisons test.

IBD-F transplantation significantly caused colitis and anxiety/depression in transplanted mice compared with HV-F transplantation. Therefore, we analyzed the gut microbiota composition of the feces of mice transplanted with IBD/D^+^-F or IBD/D^−^-F (Fig. 5, Supplementary Figures S7 and S8). The α-diversity (OUT richness), but not Shannon’s diversity index, was significantly higher in IBD-F than in HV-F (Fig. 5A). The β-diversity was also significantly different between the gut microbiota compositions of mice transplanted with HV-F and IBD-F. Compared with the bacterial composition at the phylum level, the Proteobacteria population was higher in the feces of mice transplanted with IBD-F than in those of mice transplanted with HV-F (Fig. 5B). At the family level, the *Enterococcaceae, Bacteroidaceae*, and *Prevotellaceae* populations were increased by the FMTs from IBD-F vs. HV-F. Furthermore, the *Enterococcaceae* populations were higher in the IBD/D^+^-F than in the IBD/D^−^-F (Fig. 5C). The HV-F transplantation also modified gut microbiota in the transplanted mice compared with normal control mice: it increased the populations of *Coriobacteriaceae* and *Acholeptasmataceae*, not *Enterobacteriaceae*. The FMTs from patients with IBD increased the LPS level compared with those from HV (Fig. 5D).

**Figure 6:**
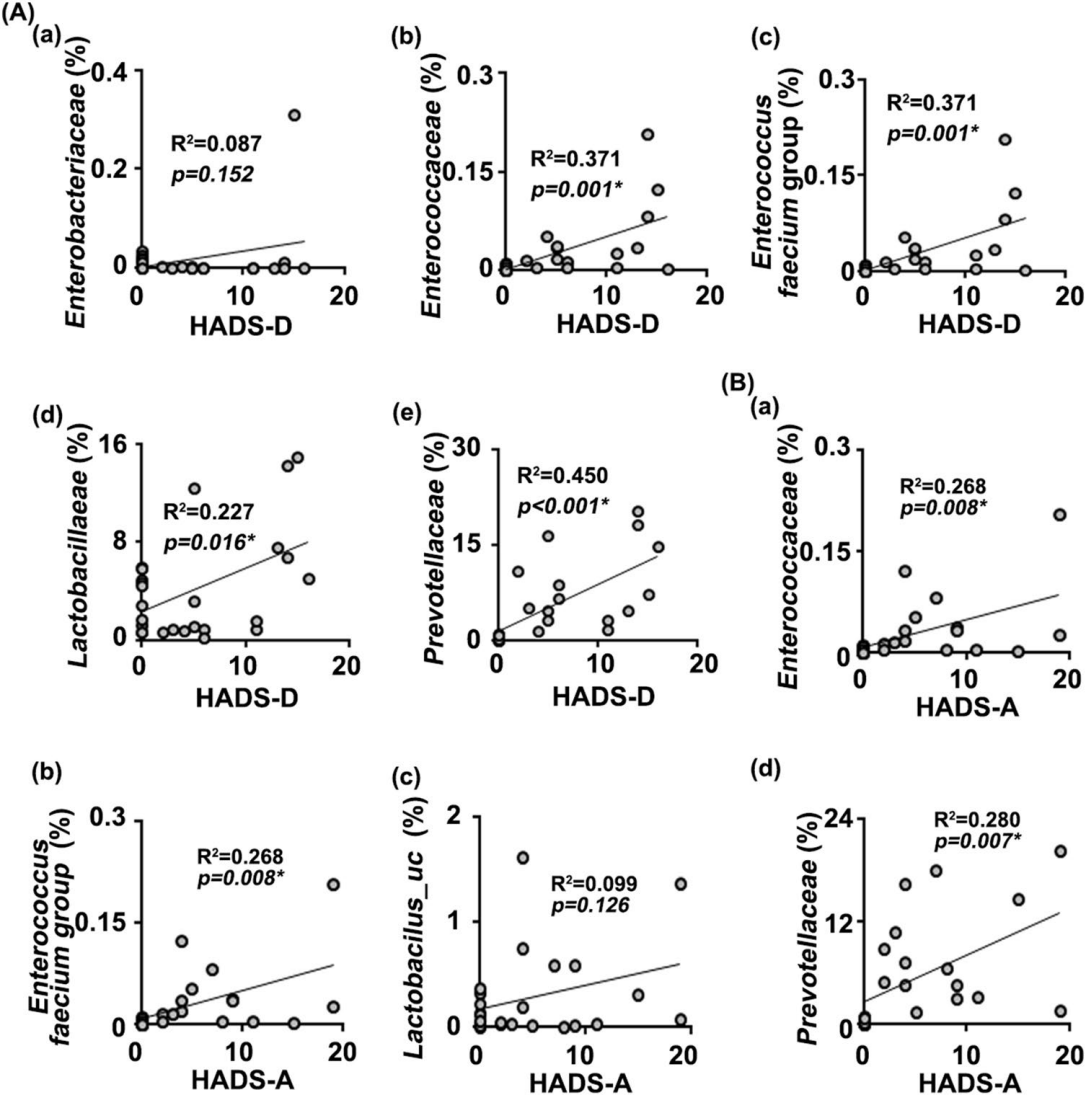
The relationship between HADS and gut microbiota composition. (**A**) The relationship between HADS-D and gut microbiota composition: (a) *Enterobacteriaceae*, (b) *Enterococcaceae*, (c) *Enterococcus faecium* group, (d) *Lactobacillaceae*, and (e) *Prevotellaceae*. (**B**) The relationship between HADS-A and gut microbiota composition: (a) *Enterococcaceae,* (b) *Enterococcus faecium* group, (c) Lactobacillus_uc, and (d) *Prevotellaceae*. n was 18 (HV n = 6; IBD/D^−^ n = 8; IBD/D^+^ n = 7). NC n = 6; HV-F n = 4; IBD/D^−^ n = 8; IBD/D^+^ n = 7: each n value is the average obtained from 3 mice.

Next, we analyzed the correlation between HADS in patients and gut microbiota composition in mice transplanted with IBD/D^+^-F or IBD/D^−^-F (Fig. 6). *Lactobacillacease* (R^2^ = 0.227, *p* = 0.016), *Enterococcaceae* (R^2^ = 0.371, *p* = 0.001), *Prevotellaceae* (R^2^ = 0.450, *p* < 0.001), and *Enterobacteriaceae* (R^2^ = 0.087, *p* = 0.152) were positively correlated with the index of HADS-D (Fig. 6A). *Enterococcaceae* (R^2^ = 0.268, *p* = 0.008) and *Prevotellaceae* (R^2^ = 0.280, *p* = 0.007) were positively correlated with the index of HADS-A (Fig. 6B). Moreover, even if outliers were excluded, the correlation between the composition of gut bacteria and depression index was not different to those included them (data not shown).

### FMT from HV alleviated IBD/D^+^-F-induced depression and colitis in mice

**Figure 7:**
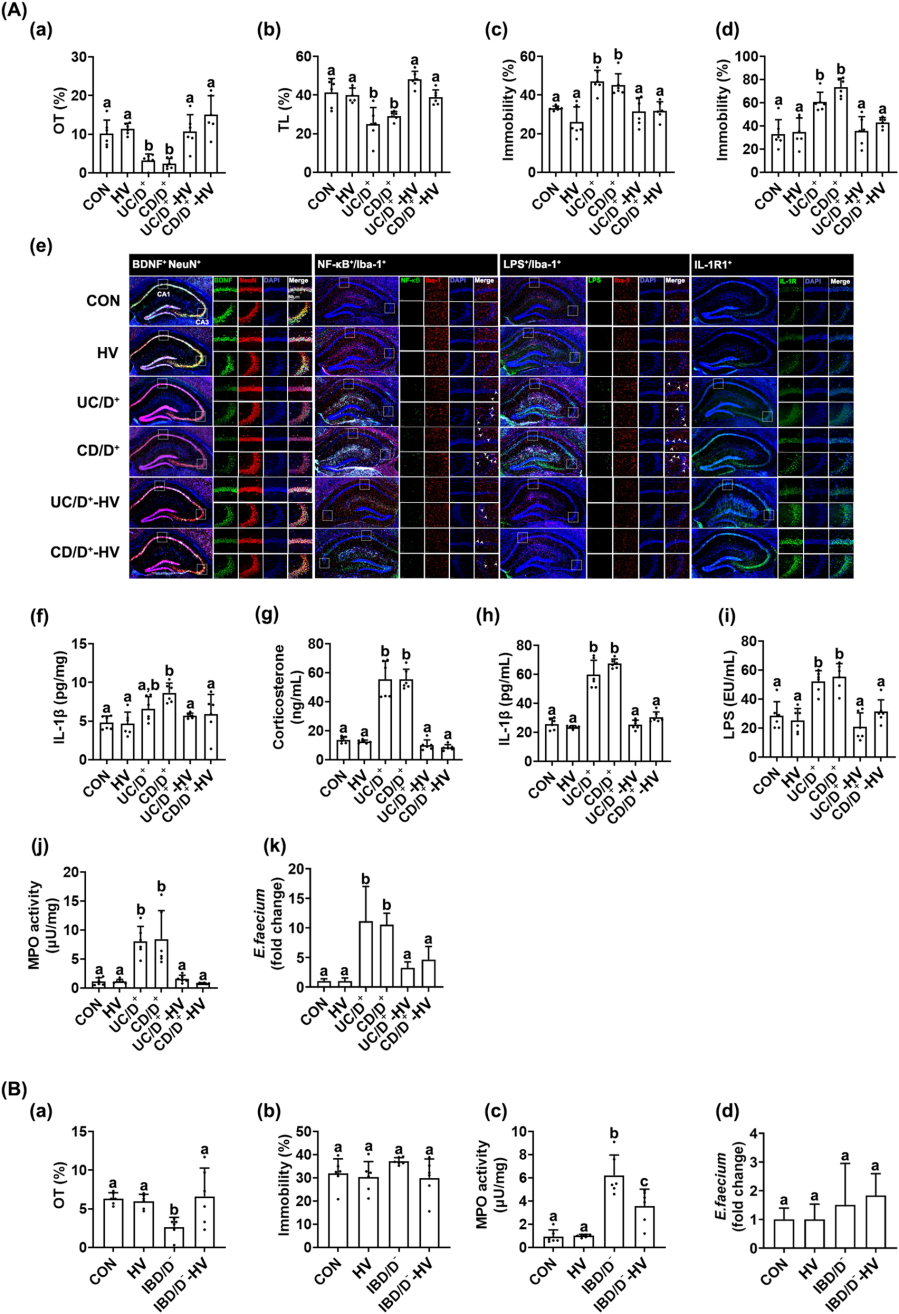
Fecal microbiota transplantation (FMT) from healthy volunteers (HVs) alleviated IBD/D^+^-F-induced depression and colitis in the transplanted mice. (**A**) Effects in IBD/D^+^-F (UC/D^+^-F- or CD/D^+^-F)-gavaged mice. Effects on the occurrence of anxiety/depression in the EPM (a) and LDT tasks (b), TST (c), and FST (d). Effects on the BDNF^+^/NeuN^+^, NF-κB^+^/Iba1^+^, LPS^+^/Iba1^+^, and IL-1R^+^ cell populations (e) and IL-1β expression (f) in the hippocampus (f). Effects on the corticosterone (g), IL-1β (h), and LPS levels (i) in the blood. (j) Effects on the myeloperoxidase (MPO) activity in the colon. (k) Effects on the fecal *Enterococcus* sp. population. (**B**) Effects in IBD/D^−^-F (CD/D^−^)-gavaged mice. Effects on the occurrence of anxiety/depression in the EPM task (a) and TST (b). (c) Effects on MPO activity in the colon. (d) Effects on the fecal *Enterococcus* sp. population. HF, IBD/D^+^-F, or IBD/D^−^-F suspension was orally transplanted in mice once a day for 5 days. Control mice (CON) were treated with vehicle (saline) instead of fecal suspension. From the next day, HV-F suspension was gavaged in IBD/D^+^-HV and IBD/D^−^-HV mice once a day for 5 days. Con, IBD/D^−^, and IBD/D^+^ mice were treated with vehicle (saline) instead of fecal suspension. Data values were indicated as mean ± SD (n = 6). Fecal Enterococcus sp. population was analyzed by using qPCR. Means with same letters are not significantly different (*p* < 0.05). All data were analyzed using ANOVA with Tukey’s multiple comparisons test.

To confirm the effects of gut microbiota in the occurrence of IBD and anxiety/depression, we prepared mice with IBD/D^+^-F- or IBD/D^−^-F-transplanted depression and colitis and transplanted the fecal microbiota of HVs (Fig. 7, Supplementary Figure S6). The FMTs from patients with IBD/D^+^ (UC/D^+^ and CD/D^+^) all caused anxiety-/depression-like behaviors and colitis. The FMTs from patients with IBD/D^−^ caused anxiety-like, but not depression-like, behaviors (Fig. 7A). However, the FMT from HV suppressed IBD/D^+^-F (UC/D^+^-F and CD/D^+^-F)-induced anxiety-like behaviors in the EPM, MB, and LDT tasks and depression-like behaviors in the TST and FST; and reduced the NF-κB^+^/Iba1^+^, LPS^+^/Iba1^+^, and IL-1R^+^ cell populations and IL-1β and IL-6 expression in the hippocampus and corticosterone, IL-6, and LPS levels in the blood, while the IBD/D^+^-F (UC/D^+^-F and CD/D^+^-F)-suppressed BDNF^+^/NeuN^+^ cell population increased. The FMT from HV suppressed colon shortening, reduced myeloperoxidase activity, and IL-6 expression in the colon of mice with the IBD/D^+^-F-induced depression. Furthermore, the FMT from HV significantly reduced the IBD/D^+^-F-induced *Enterococcus* sp. population and bacterial LPS production in the feces.

The FMT form HV also alleviated the IBD/D^−^-F-induced anxiety-like behavior, IL-6 expression in the hippocampus, corticosterone, LPS, and IL-6 levels in the blood, and colon shortening, myeloperoxidase activity, and IL-6 expression in the colon (Fig. 7B).

## Discussion

Gut microbiota is closely involved in the occurrence of psychiatric disorders through the activation of the MGB axis^29^. Many studies have focused on the role of gut microbiota in the outbreak of neuropsychiatric disorders^29–31^. *Klebsiella* and *Lachnospira*, which belong to the Firmicutes phylum, are higher, while the *Escherichia* and *Bifidobacterium* populations are lower, in the feces of patients with depression than in the feces of healthy individuals^32^. The *Enterobacteriacea*e and *Alistipes* populations are over-represented in patients with depression^32^. The induction of depression by stressors triggers colitis and increases the gut Proteobacteria population and bacterial LPS production in mice^11,12^. The FMT from stress-stimulated mice also causes colitis with depression in the transplanted mice^11^.

The fecal microbiota transplantation (FMT) from IBD patients causes the IBD-like colitis in germ-free mice^26,27^. The FMT from Chrna7 KO mice causes depression-like phenotypes and systemic inflammation in antibiotics-treated mice via the subdiaphragmatic vagus nerve^33^. The FMT from mice with colitis occurs colitis as well as depression in conventional mice^11^. The FMT from NLRP3-deficient mice alleviates depressive-like behaviors by regulating astrocyte dysfunction in antibiotics-treated mice^34^. The FMT from healthy individuals alleviates IBD in clinic studies^27,28^. These findings suggest that the microbiota are closely connected with the occurrence of IBD and neuropsychiatric disorders, such as depression, through the microbiota − gut − brain (MGB) axis. Several studies have demonstrated that the high prevalence of commensal gut Proteobacteria including *Enterobacteriaceae* is closely associated with IBD, including UC and CD, overexpression of gut bacterial LPS^25^. However, the character of gut microbiota associated with the occurrence of IBD and depression is not sufficiently understood.

In the present study, the FMTs from patients with IBD caused colitis and depression in the transplanted mice: they induced the NF-κB^+^/Iba1^+^, LPS^+^/Iba1^+^ and IL-1R^+^ cell populations and IL-1β and IL-6 expression in the hippocampus and myeloperoxidase activity and IL-1β and IL-6 expression and suppressed tight junction protein expression in the hippocampus and colon. After the segregation of patients with IBD/D^−^ and IBD/D^+^, the FMTs from patients with IBD/D^−^ or IBD/D^+^ all caused colitis and anxiety in the transplanted mice, while the FMTs from patients with IBD/D^+^, but not IBD/D^−^, caused depression. Parsing anxiety-like behaviors is different from depressive behaviors in humans^35^. The high rate of patients with anxiety disorder has symptoms of depression. Therefore, evaluating anxiety- and depression-like behaviors in mice may be not sufficient to understand the multiple etiologies of patients with anxiety and/or depression^35^. Nevertheless, it is helpful to understand the physiological effects of gut microbiota on the occurrence of anxiety and/or depression through the activation of MGB axis activation. These findings suggest that gut microbiota are closely involved in the occurrence of anxiety and depression.

FMTs from patients with IBD/D^+^ or IBD/D^−^ also induced myeloperoxidase activity and IL-1β and IL-6 expression in the colon. The FMTs from patients with IBD/D^+^ increased the stenosis score and decreased tight junction protein expression more strongly than did the FMTs of patients with IBD/D^−^. However, after the segregation of patients with UC and CD, the FMTs from patients with UC and CD caused colitis with anxiety/depression in the transplanted mice. Interestingly, the occurrence of colon stenosis was observed at a greater extent in mice transplanted with the feces of CD patients than in those transplanted with the feces of patients with UC, as patients with IBD^36^. These results suggest that the gut microbiota consists of IBD-inducing, -suppressing, and -irrelevant microbes and that some IBD-inducing microbes may cause anxiety with or without depression.

We also found that the gut microbiota composition of IBD-F was significantly different from that of HV-F: the α-/β-diversities of IBD-F were lower than those of HV-F, and the β-diversity was different between IBD-F and HV-F, as reported previously^37–39^. Although the α-diversity was not significantly different between IBD/D^−^-F and IBD/D^+^-F, the β-diversity was different. IBD-F exhibited a higher abundance of the Proteobacteria population compared with HV-F, while the Bacteroidetes population was lower in IBD-F. In particular, IBD/D^+^-F exhibited a higher abundance of *Enterobacteriaceae, Enterococcaceae*, and *Lactobacillaceae* compared with IBD/D^−^-F. Among these gut bacteria, the Enterobacterales_f and *Enterococcus* populations were positively correlated with the depression index in patients with IBD (HADS-D) and the Enterobacterales_f, Enterobacterales_g, *Alistipes*, and Enterobacterales group populations were positively correlated with the anxiety index in patients with IBD (HADS-A). These results suggest that the IBD with anxiety/depression may be induced in the intestine by *Enterobacteriaceae, Enterococcaceae*, and *Lactobacillaceae*.

The fecal microbiota compositions of mice transplanted with HV-F and IBD-F partially matched those of patients with HV-F and IBD, respectively. However, those of mice transplanted with HV-F was significantly different to those of mice transplanted with IBD and normal control mice. The Proteobacteria population, including *Enterococcaceae*, was increased in the feces of mice transplanted with IBD-F than it was in those transplanted with HV-F, similar to those of IBD-F itself. The *Enterococcaceae, Lactobacilliaceae,* and *Enterobacteriaceae* populations were positively correlated with the anxiety/depression index of patients with IBD (HADS-A and HADS-D). However, the *Bacteroidaceae, Prevotellaceae,* and *Helicobacteraceae* populations were increased in the feces of mice transplanted with IBD-F compared with mice transplanted with HV-F. Jang et al. reported that the FMTs from mice with or without depression shifted the gut microbiota composition, which persisted for more than 15 days in specific pathogen-free mice^11^. Lee et al. reported that the incidence of cognitive impairment by exposure to a human gut bacterium, *Paenalcaligenes hominis*, was severe 10-fold in germ-free mice more than in SPF mice^21^. These results support the suggestion that the gut microbiota of IBD-F comprises IBD-inducing microbiota, such as *Enterobacteriaceae* and *Enterococacceae*, which may be transient and attachable in the gastrointestinal tract.

The content of LPS was higher in the IBD-F than in the HV-F. Furthermore, the fecal and blood LPS levels were higher in IBD-F-gavaged mice than they were in HV-F-gavaged mice. The FMTs from IBD patients significantly suppressed tight-junction protein expression in the colon and hippocampus. They also suppressed BDNF^+^/NeuN^+^ cell population in the hippocampus, while the NF-κB^+^/Iba1^+^, LPS^+^/Iba1^+^ and IL-1R^+^ cell populations and IL-1β and IL-6 expression increased. Among the IBD-F, the levels of IBD markers such as IL-1β , IL-6, LPS, and NF-κB^+^/Iba1^+^ cells were induced more strongly by IBD/D^+^-F than by IBD/D^−^. Exposure to *Escherichia coli* increases the hippocampal NF-κB^+^/Iba1^+^ and LPS^+^/Iba1^+^ cell populations and blood and fecal LPS levels and decreases the hippocampal BDNF^+^/NeuN^+^ cell population and tight-junction protein expression, leading to depression^11,37^. Oral gavage of *Escherichia coli* or LPS causes colitis and neuroinflammation in mice by suppressing tight-junction protein expression^21^. An IL-1R antagonist mitigates depression^40^. Exposure to stressors such as high-fat diet and ampicillin increases the Proteobacteria population and gut bacterial LPS production^11,41,42^. They induce gastrointestinal inflammation and suppress tight-junction protein expression, leading to a leaky gut, which accelerates the translocation of gut bacteria and their by-products, such as LPS, across the intestinal mucosa. In the present study, we found that the transplantation of IBD-F significantly increased the LPS levels in the blood and feces and caused neuroinflammation with colitis in mice compared with HV-F transplantation. Moreover, IBD patients suffer from leaky gut^43^. Excessive absorption of LPS across the leaky gut can cause systemic inflammation, leading to the neuroinflammation in the brain^44,45^. In the present study, we found that anxiety-/depression-like behaviors and their related markers BDNF expression and NF-κB activation were more severely fluctuated in the FMTs from patients with IBD/D^+^ than in ones from patients with IBD/D^−^. These results suggest that the FMT from patients with IBD/D^+^ may cause colitis with a leaky gut, which may accelerate the absorption of fecal LPS into the blood and suppress the NF-κB-mediated BDNF expression in the brain, resulting in the occurrence of anxiety/depression through the regulation of neuroinflammation.

However, the FMT from HV suppressed IBD/D^+^-F (UC/D^+^-F and CD/D^+^-F)-induced anxiety- and depression-like behaviors, NF-κB^+^/Iba1^+^, LPS^+^/Iba1^+^, and IL-1R^+^ cell populations, hippocampal IL-1β and IL-6 expression in the hippocampus, blood corticosterone, IL-6, and LPS levels, and colonic myeloperoxidase and IL-6 expression in mice, while the IBD/D^+^-F (UC/D^+^-F and CD/D^+^-F)-suppressed BDNF^+^/NeuN^+^ cell population increased in the hippocampus. Furthermore, the FMT from HV significantly reduced the IBD/D^+^-F-induced *Enterococcus* sp. population and bacterial LPS production in the feces. He et al. reported that the FMT from HV prevented the relapse of seizures and CD activity score after withdrawing the antiepileptic drugs in a patient with CD and epilepsy^46^. Goo et al. reported that the FMT for normal control mice alleviated cognitive deficits and social withdrawal symptoms observed in autism spectrum disorders in Fmr1 KO mice with autistic-like behaviors by increasing the population of *Akkermansia muciniphila*^47^. These results support the hypothesis that gut microbiota are closely connected with the occurrence of IBD and neuropsychiatric disorders, such as depression, by the regulation of the MGB axis activation.

In conclusion, the outbreak of depression/anxiety may be dependent on the systemic inflammation with a leaky gut through the gut dysbiosis-attributable overproduction of bacterial LPS and suppression of tight junction protein expression in patients with IBD..

## Materials and methods

### Materials

A De Man, Rogosa, and Sharpe (MRS) medium and Sabouraud dextrose agar (SDA) were purchased from BD (Franklin Lakes, NJ). An antibody for NF-κB was purchased from Cell Signaling Technology (Danvers, MA). Antibodies for CD11c, Iba1, and BDNF were purchased from Abcam (Cambridge, U.K.). Alexa Fluor 488 and Alexa Fluor 594 were purchased from Invitrogen (Carbsband, CA). Enzyme-linked immunosorbent assay (ELISA) kits for TNF-α, IL-6, and IL-10 were purchased from Ebioscience (Atlanta, GA). A QIAamp Fast DNA stool mini kit was purchased from Qiagen (Hilden, Germany).

### Volunteers

Volunteers, HVs (average age, 36.5 ± 8.8 years) and patients with IBD/D^−^ (average age, 36.0 ± 12.6 years) and IBD/D^+^ (average age, 46.4 ± 15.3 years), were recruited from Kyung Hee University (Seoul, Korea) (Supplementary Table S1). They are not a vegetarian or a meatarian. The stools were collected from volunteers, if antibacterial medications had not been administered within 3 month. Furthermore, HVs had not received analgesics and anti-inflammatory drugs within 3 months. However, most of IBD patients received steroids, immuno-modulators, such as azathioprine or methotrexate, and/or biologics/small molecules for therapeutic purpose. All patients with IBD enrolled in the study were > 13 years of age at the diagnosis of IBD. All diagnoses were confirmed by previously established international criteria based on clinical, endoscopic, histopathological, and radiological findings^48^. Psychometric tools (Patient health questionnaire-9 [HQ-9] and hospital and anxiety depression scale [ADS] were assessed according to the method of Song et al.^49^ The study protocol and consent forms for the stool collection were approved by the Committee for the Care and Use of Clinical Study of the Medical School of Kyung Hee University (IRB File No., KHUH 2018-03-006-018 and KHUH 2018-12-004-003). All participants agreed to participate in the study and signed informed consent before the initiation of the study. In a patient aged under 18 (n = 1), informed consent was obtained from a parent. All experiments related to the usage of human feces were conducted in compliance with the principles of the Declaration of Helsinki and the Korean Good Clinical Practice guidelines.

### Animals

C57BL/6 mice (male, 6 weeks old, 19–21 g) were purchased from Koatech Inc. (Seoul, Korea). Mice were maintained in plastic cages with the 5-cm raised wire floor, which was designed to protect mice for feeding the feces, under a controlled condition (temperature, 20^○^C–22^○^C; humidity, 50% ± 10%; light/dark cycle, 12-h) and fed standard laboratory chow and water ad libitum. Mice were acclimatized for 1 week before the use of experiments. All animal experiments were approved by the Institutional Animal Care and Use Committee of Kyung Hee University (IACUC No., KUASP(SE)-18-045, KUASP(SE)-19-290, and KHSASP-20-078] and were performed according to the NIH, AAALAC International, and University Guide for Laboratory Animals Care and Usage. This study additionally adheres to standards articulated in the ARRIVE guidelines.

### Transplantation of fecal microbiota suspension into mice

For the preparation of fecal microbiota suspension, the feces of patients IBD/D^−^ (IBD/D^−^-F, n = 8), patients with IBD/D^+^ (IBD/D^+^-F, n = 7), or HVs (HV-F, n = 6) were freshly collected, immediately (< 2 h) suspended in saline, filtrated through sterilized gauze and centrifuged at 500 g at 4 °C for 5 min. The supernatant fraction (10 mg [wet weight]/kg/day: IBD/D^−^-F, n = 8); IBD/D^+^-F, n = 7; HV-F, n = 4 [not sufficiently collect the feces in two HVs]) was used for FMT experiments.

First, to understand the effects of gut microbiota on the occurrence of depression and colitis, each fecal microbiota suspension of the IBD patients or HVs (10 mg feces [wet weight]/kg/day) was orally gavaged into three mice once a day for 5 days, respectively.

Second, to understand the anti-depressive effects of gut microbiota, the fecal microbiota suspension of patients with IBD/D^+^ was orally gavaged into twelve mice once a day for 5 days. From next day after the final gavage of the fecal microbiota suspension of patients with IBD/D^+^, the fecal microbiota suspension from HVs (10 mg feces [wet weight]/kg/day) or vehicle was orally gavaged once a day for 5 day.

Anxiety-/depression-like behaviors were measured on the next day following treatment with the fecal or bacterial suspension^11,37^. Twelve hours after the final behavioral task, mice were euthanized by the inhalation of CO^2^. Colons and brains were removed for the enzyme-linked immunosorbent assay (ELISA) and immunoblotting assay from mice and stored at -80 °C for ELISA and immunoblotting. Colons and brains were removed for the immunofluorescence assay from mice that were transcardially perfused with 4% paraformaldehyde.

### Behavioral tasks

The elevated plus maze (EPM) task was assessed in a plus-maze apparatus (consisting of two enclosed [30 × 7 cm] and two open [30 × 7 cm] arms with 20-cm-high walls extending from a central platform [7 × 7 cm]), as previously reported^11^. The light/dark transition (LDT) task was assessed in a light/dark box apparatus (45 × 25 × 25 cm, consisting of two chambers made of black and white Plexiglass [floor] and polywood [walls] connected by an opening [7.5 × 7.5 cm] located at floor level in the center of the dividing wall), as previously reported^11^. The marble burying (MB) task was assessed in a smooth, opaque plastic cage (30 × 35 × 13 cm) with a 5-cm layer of sawdust and 30 marbles on top of it (five rows or marbles regularly spaced at 2-cm away from the walls), as previously reported^11^. The number of marbles buried for 30 min was counted. The tail suspension test (TST) was assessed on the edge of a table, at 30-cm above it, for 5 min, as previously reported^35^. Mice were judged to be immobile, when they did not move and hung passively. The forced swimming test (FST) was assessed in a round transparent plastic jar (20 × 40 cm^3^) containing fresh water (25 °C) to a height of 25-cm for 5 min, as previously reported^37^. Mice were judged to be immobile, when they remained floating in the water without struggling.

### Immunofluorescence assay

The brains and colons were post-fixed with paraformaldehyde, cytoprotected in 30% sucrose solution and cryosectioned. Sectioned tissues were immunostained (Supplementary Methods) and observed by a confocal microscope, as reported previously^21^.

### ELISA and immunoblotting

Brain and colon tissues were homogenized with radioimmunoprecipitation assay lysis buffer (Biosesang Inc., Seongnam, Korea) containing a protease inhibitor cocktail and a phosphatase inhibitor cocktail at 4 °C. Cytokine levels were determined in their supernatants using ELISA kits (eBioscience, San Diego, CA) according to the method of Jang et al.^11^ Corticosterone, IL-1β and IL-6 levels were measured using ELISA kits^11^.

### Limulus amebocyte lysate (LAL) and myeloperoxidase activity assays

Fecal and blood endotoxin levels were assayed using an LAL assay kit (Cape Cod Inc., E. Falmouth, MA) according to the method of Jang et al.^11^ Colonic myeloperoxidase activity was assayed according to the method of Jang et al.^11^.

### Pyrosequencing

Bacterial genomic DNAs were extracted from HV-F, IBD-F, and mouse feces using a QIAamp DNA stool mini kit^37^. Amplification of genomic DNA was carried out using the bacterial 16S rRNA V4 region gene-targeted barcoded primers and sequenced using Illumina iSeq 100 (San Diego, CA). Data availability 16S sequencing dataset (pyrosequencing reads) was deposited in the NCBI’s short read archive under accession number PRJNA666980.

### Statistics

Experimental data are described as the mean ± SD using GraphPad Prism 8 (GraphPad Software, Inc., San Diego, CA, USA). Significant differences were analyzed using one or two-tailed Mann–Whitney U test for non-parametric test, unpaired *t* test, ANOVA and Tukey's multiple comparisons test, one-way ANOVA with post-hoc Bonferroni's comparisons test (*p* < 0.05).

### Ethics statement

The study protocol and consent forms for the human stool collection were approved by the Committee for the Care and Use of Clinical Study of the Medical School of Kyung Hee University (IRB File No., KHUH 2018-03-006-018 and KHUH 2018-12-004-003). All experiments were conducted in compliance with the principles of the Declaration of Helsinki and the Korean Good Clinical Practice guidelines.

All animal experiments were approved by the Institutional Animal Care and Use Committee of Kyung Hee University (IACUC No., KUASP(SE)-18-045, KUASP(SE)-19-290, and KHSASP-20-078] and were performed according to the NIH, AAALAC International, and University Guide for Laboratory Animals Care and Usage.

## Supplementary Information

Supplementary Information.
