# Supplementary material for: Transplantation of fecal microbiota from patients with inflammatory bowel disease and depression alters immune response and behavior in recipient mice
Source: Sci Rep. 2021 Oct 14;11:20406. doi: 10.1038/s41598-021-00088-x (PMC8516877; doi:10.1038/s41598-021-00088-x)
Supplement: Supplementary file 1 — Supplementary Information. [file 41598_2021_88_MOESM1_ESM.docx]

[Supplementary Information]

**Transplantation of fecal microbiota from patients with inflammatory bowel disease and depression alters immune response and behavior in recipient mice**

Hyo-Min Jang^a,#^, Jeon-Kyung Kim^a,#^, Min-Kyung Joo^a,#^, Yun-Jeong Shin^a^, Chang Kyun Lee^b^, Hyo-Jong Kim^b^, Dong-Hyun Kim^a,*^

*^a^Neurobiota Research Center, College of Pharmacy, Kyung Hee University, 26, Kyungheedae-ro, Dongdaemun-gu, Seoul 02447, Korea; ^b^Department of Internal Medicine, Kyung Hee University School of Medicine, Seoul 02447, Korea*

**Methods**

**Immunofluorescence assay**

The brains and colons were removed from mice perfused and post-fixed with paraformaldehyde, cytoprotected in 30% sucrose solution and cryosectioned. Sectioned tissues were immunostained according to the method of Kim et al. [1]. Briefly, the sections were washed with phosphate-buffered saline, blocked with normal serum and incubated with antibodies against NF-κB (p-p65, 1:100, Cell Signaling Technology: cat # 3033S), LPS (1:100, Abcam: cat #ab35654), NeuN (1:200, Millipore: cat #MAB377), Iba1 (1:200, Thermo Fisher Scientific: cat #PA5-27436), BDNF (1:50, Santa Cruz Biotechnology: cat # SC-65513), IL-1R (1:100, Abcam: cat #ab106278) and/or CD11c (1:100, Abcam: cat #ab11029) overnight, followed by incubation with secondary antibodies conjugated with Alexa Fluor 594 (1:200, Invitrogen) or Alexa Fluor 488 (1:200, Invitrogen) for 2 h. Cell nuclei were stained with 4′,6-diamidino-2-phenylindole, dilactate (Sigma Aldrich: cat #F6057). Immunostained sections were observed using a confocal laser microscope.

**References**

1. Kim JK, Lee KE, Lee SA, Jang HM, Kim DH. Interplay between human gut bacteria Escherichia coli and Lactobacillus mucosae in the occurrence of neuropsychiatric disorders in mice. Front Immunol. 2020;11: 273.

**Table S1.** Clinical characteristics of study participants

(A)

| Group | | Age  (year of birth) | Sex^1)^ | Psychiatric disorders^2)^ | | Disease activity | Location^3)^ | Medication^4)^ |
| --- | --- | --- | --- | --- | --- | --- | --- | --- |
|  |  |  |  | HADS-A | HADS-D |  |  |  |
| Healthy volunteers (HVs) | | 39 (1979) | F | N/A | N/A | None | None | N/A |
|  |  | 37 (1982) | F | N/A | N/A | None | None | N/A |
|  |  | 41 (1978) | F | N/A | N/A | None | None | N/A |
|  |  | 36 (1938) | F | N/A | N/A | None | None | N/A |
|  |  | 20 (1998) | M | N/A | N/A | None | None | N/A |
|  |  | 46 (1972) | F | N/A | N/A | None | None | N/A |
| Mean±SD | | 36.5±8.8 |  | - | - |  |  | - |
| Patients with IBD/D^− 5)^ | UC | 42 (1977) | F | 4 | 5 | Active | - | B |
|  |  | 34 (1984) | M | 2 | 6 | Active | U2 | B |
|  |  | 51 (1967) | M | 4 | 5 | Inactive | U1 | B |
|  |  | 48 (1971) | M | 9 | 5 | Inactive | U1 | None |
|  |  | 44 (1976) | M | 8 | 6 | Active | U2 | B,C |
|  | CD | 30 (1990) | M | 2 | 3 | Inactive | C2 | A,B |
|  |  | 14 (2006) | F | 3 | 2 | Active | C1 | C |
|  |  | 25 (1994) | M | 5 | 4 | Severe active | C2 | B,C |
| Mean±SD | | 36.0±12.6 |  | 4.6±2.6 | 4.5±1.4 |  |  |  |
| Patients with IBD/D^+ 6)^ | UC | 59 (1959) | M | 15 | 16 | Inactive | U1 | None |
|  |  | 57 (1962) | M | 9 | 13 | Inactive | U1 | B |
|  |  | 64 (1955) | M | 11 | 11 | Active | U2 | C |
|  |  | 26 (1993) | F | 19 | 11 | Active | U1 | A,B |
|  | CD | 26 (1992) | F | 19 | 14 | Inactive | C3 | C |
|  |  | 46 (1972) | M | 7 | 14 | Severe active | C2 | B,C |
|  |  | 47 (1971) | M | 4 | 15 | Inactive | C3 | C |
| Mean±SD | | 46.4±15.3 |  | 12.0±5.9 | 13.4±1.9 |  |  | - |

^1)^ Gender information was described as M (Male) and F (Female).

^2)^ Psychiatric disorders were described as HADS (Hospital anxiety and depression scale) A (anxiety) and D (depression) score.

^3)^ Classification according to the location of the lesion. In patients with ulcerative colitis, U1 means ulcerative proctitis confined to the rectum and U2 stands for Left sided (distal) ulcerative colitis under the splenic flexure. It can be pan-gastroenterological in patients with Crohn's disease, but as a characteristic location, C1; ileal, C2; colonic and C3; ileocolonic.

^3)^ In the case of the healthy control group, it was investigated whether medicines that can affect the gut microbiota, such as analgesics, anti-inflammatory drugs, and antibiotics, were administered within 3 months. And in the case of patients, medications were expressed in A (Steroid), B (Immuno-modulators, such as azathioprine or methotrexate) and C (Biologics/small molecules) for therapeutic purpose.

^5)^ Inflammatory bowel disease patients without depression (score, <10 on HADS-D)

^6)^ Inflammatory bowel disease patients with depression (score, >10 on HADS-D)

(B)

| Group | | Age  (year of birth) | Sex | Height (m) | Weight (kg) | Body mass index^1)^ | Lab finding^2)^ | | | | |
| --- | --- | --- | --- | --- | --- | --- | --- | --- | --- | --- | --- |
|  |  |  |  |  |  |  | WBC (10^3^/μL) | Hb (g/dL) | Albumin (g/dL) | ESR (mm/hr) | CRP (mg/dL) |
| Healthy volunteers (HVs) | | 39 (1979) | F | 1.65 | 53.6 | 19.7 | 3.81 | 12.6 | 4.4 | - | - |
|  |  | 37 (1982) | F | 1.48 | 58.6 | 26.7 | 4.61 | 9.9 | 3.8 | - | - |
|  |  | 41 (1978) | F | 1.60 | 52.6 | 20.6 | 5.35 | 13 | 4.6 | - | - |
|  |  | 36 (1938) | F | 1.58 | 54 | 21.6 | 5.55 | 12.5 | 4.5 | - | - |
|  |  | 20 (1998) | M | 1.80 | 80.7 | 24.9 | 6.98 | 15.8 | 4.5 | - | - |
|  |  | 46 (1972) | F | 1.67 | 57.2 | 20.6 | 5.9 | 12.4 | 4.4 | - | - |
| Mean±SD | | 36.5±8.8 |  | 1.63  ±0.11 | 59.45  ±10.7 | 22.35  ±2.8 | 5.37  ±1.09 | 12.7  ±1.88 | 4.37  ±0.29 |  |  |
| Patients with IBD/D^− 4)^ | UC | 42 (1977) | F | 1.61 | 46.6 | 18.0 | 3.21 | 13.4 | 3.8 | 24 | 0.25 |
|  |  | 34 (1984) | M | 1.74 | 79 | 26.1 | 4.16 | 15.5 | 4.5 | 2 | 1.8 |
|  |  | 51 (1967) | M | 1.8 | 79 | 24.4 | 6.19 | 15.6 | 4.1 | 2.4 | 0.25 |
|  |  | 48 (1971) | M | 1.78 | 76 | 24.0 | 3.92 | 16.7 | 4.7 | N/A | 0.25 |
|  |  | 44 (1976) | M | 1.75 | 73 | 23.8 | 4.59 | 12.7 | 4.5 | 28 | 0.53 |
|  | CD | 30 (1990) | M | 1.68 | 52 | 18.4 | 10.86 | 15.7 | 4.7 | 46 | 0.25 |
|  |  | 14 (2006) | F | 1.62 | 55 | 21.0 | 10.2 | 11.6 | 3.5 | 111 | 0.84 |
|  |  | 25 (1994) | M | 1.77 | 65 | 20.7 | 7.08 | 14.9 | 4.8 | 9 | 0.25 |
| Mean±SD | | 36.0  ±12.6 |  | 1.70  ±0.1 | 65.7  ±13.0 | 22.1  ±3.0 | 6.28  ±2.91 | 14.51  ±1.75 | 4.33  ±0.47 |  |  |
| Patients with IBD/D^+ 5)^ | UC | 59 (1959) | M | 1.73 | 60 | 20.0 | 3.66 | 12.1 | 4.5 | N/A | 0.25 |
|  |  | 57 (1962) | M | 1.58 | 53 | 21.2 | 7.77 | 16 | 4.4 | 11 | 0.25 |
|  |  | 64 (1955) | M | 1.68 | 60 | 21.3 | 4.46 | 15 | 4.6 | N/A | 0.25 |
|  |  | 26 (1993) | F | 1.62 | 56 | 21.3 | 10.48 | 13.1 | 4.2 | N/A | 0.25 |
|  | CD | 26 (1992) | F | 1.68 | 58 | 20.5 | 8.66 | 13 | 4.4 | 9 | 0.25 |
|  |  | 46 (1972) | M | 1.77 | 57 | 18.2 | 14.72 | 9.6 | 2.9 | N/A | 0.25 |
|  |  | 47 (1971) | M | 1.68 | 60 | 21.3 | 3.76 | 12.4 | 4.6 | 3 | 0.25 |
| Mean±SD | | 46.4  ±15.3 |  | 1.70  ±0.1 | 57.7  ±2.6 | 20.5  ±1.2 | 7.64  ±4.09 | 13.03  ±2.07 | 4.23  ±0.60 |  |  |

^1)^ Body mass index (BMI) BMI is defined as the body mass divided by the square of the body height, and is universally expressed in units of kg/m^2^, resulting from mass in kilograms and height in meters.

^2)^ This is a simple laboratory test result, showing complete blood count (CBC) and blood chemistry tests showing white blood cells (WBC), hemoglobin (Hb), albumin, erythrocyte sedimentation rate (ESR) and C-reactive protein (CRP) levels.

**
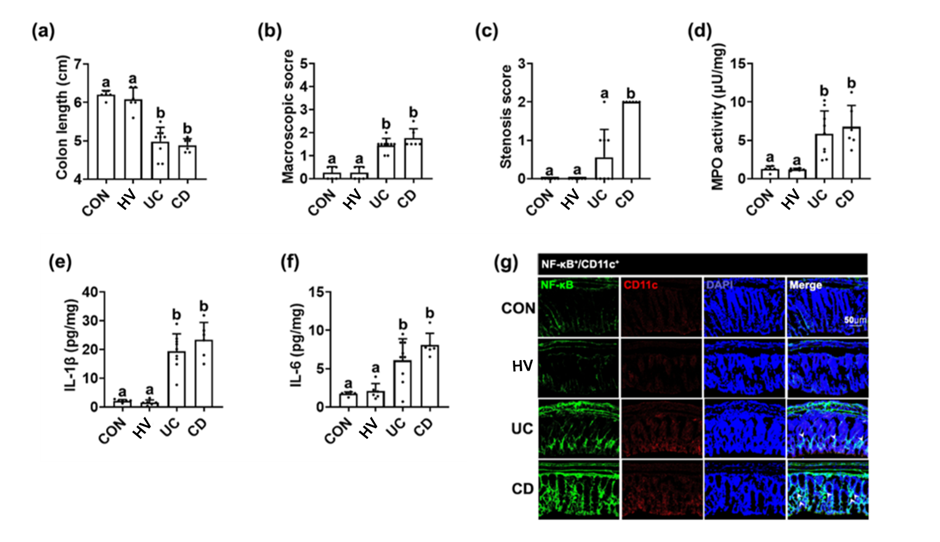
**

**Figure S1**. Fecal microbiota transplantation from patients with UC or CD or healthy volunteers (HVs) caused colitis in the transplanted mice. Effects on the colon length (a), macroscopic score (b), stenosis score (c), myeloperoxidase activity (d), IL-1β (e), and IL-6 expression (f), and NF-κB^+^/Iba1^+^ cell population (g) in the colon. Each HV-F (n=6), UC-F (n=9), or CD-F (n=6) was orally transplanted in three mice once a day for 5 days. Control mice were treated with vehicle (saline) instead of fecal suspension. Data values were indicated as mean ± SD (NC n=6; HV-F n=6; UC n=9; CD n=6: each n value is the average obtained from 3 mice). Means with same letters are not significantly different (p < 0.05). All data were analyzed using ANOVA with Tukey’s multiple comparisons test.


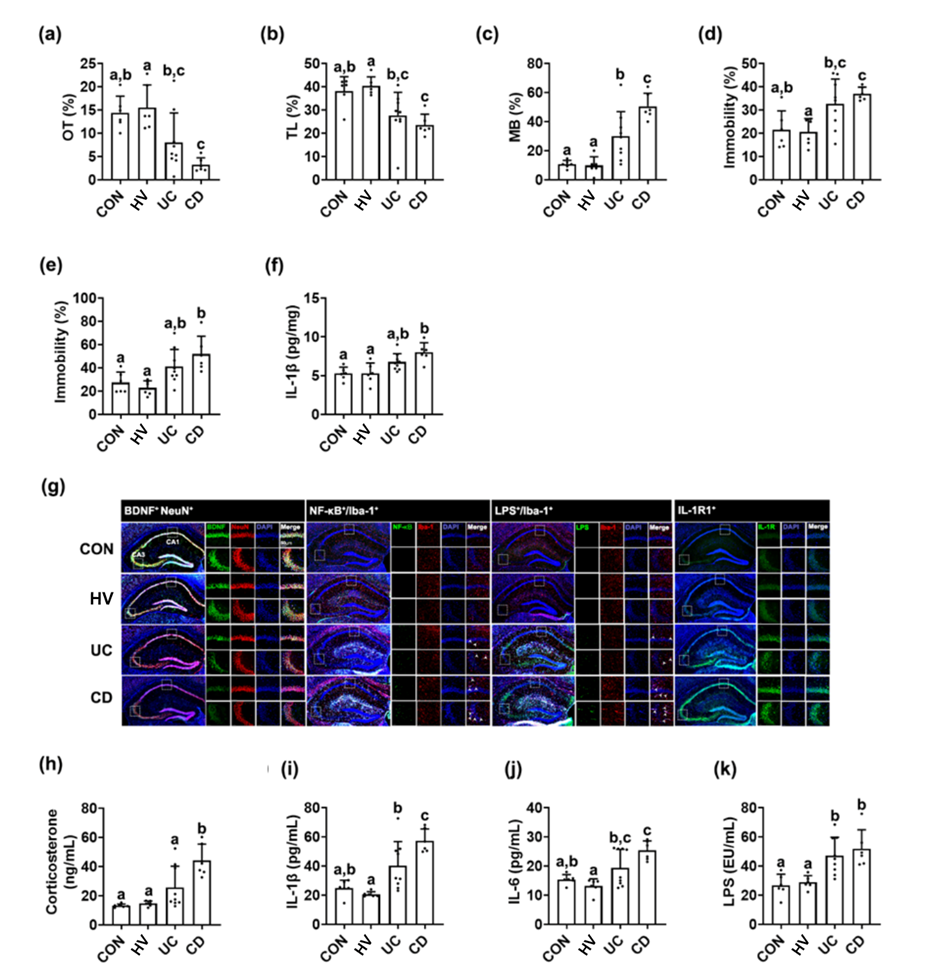


**Figure S2**. Fecal microbiota transplantation from patients with UC or CD or healthy volunteers (HVs) caused anxiety/depression in the transplanted mice. Effects on the occurrence of anxiety/depression in the EPM (a), LDT (b), MB tasks (c), TST (d), and FST (e). (B) Effects on the IL-1β expression (f), BDNF^+^/NeuN^+^, NF-κB^+^/Iba1^+^, LPS^+^/Iba1^+^ and IL-1R^+^ cell populations in the hippocampus (g). Effects on the corticosterone (h), IL-1β (i), IL-6 (j), and LPS levels (k). Each HV-F (n=6), UC-F (n=9), or CD-F (n=6) was orally transplanted in three mice once a day for 5 days. Control mice were treated with vehicle (saline) instead of fecal suspension. Data values were indicated as mean ± SD (NC n=6; HV-F n=6; UC n=9; CD n=6: each n value is the average obtained from 3 mice). Means with same letters are not significantly different (p < 0.05). All data were analyzed using ANOVA with Tukey’s multiple comparisons test.


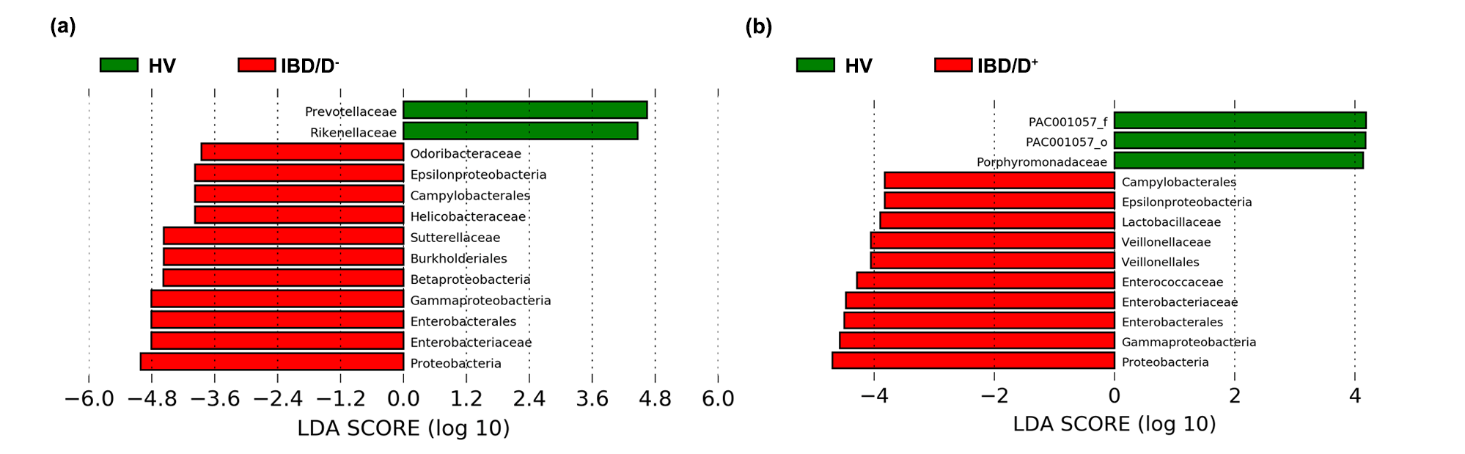


**Figure S3.** The fecal microbiota composition of patients with IBD/D^−^ or IBD/D^+^ and healthy volunteers (HVs). Effects on the linear discriminant analysis (LDA) in Galaxy (http://huttenhower.sph.harvard.edu/galaxy/) between HV and patients with IBD/D^+^. PAC001057 is belong to Mollicutes. The gut microbiota composition was analyzed by using using Illumina iSeq 100. Data values were indicated as mean ± SD (HV n=6; IBD/D^−^ n=8; IBD/D^+^ n=7).


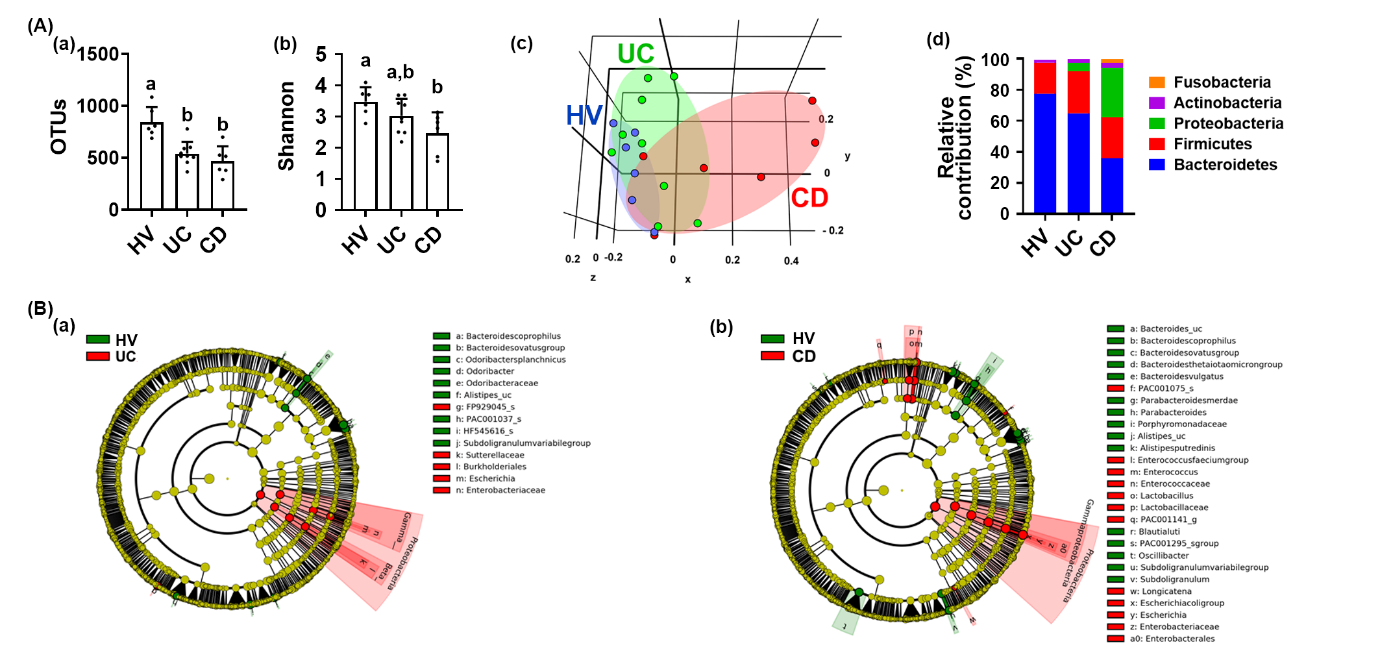


**Figure S4.** The fecal microbiota composition of patients with UC or CD and healthy volunteers (HVs). (A) Effects on OTU richness (a), Shannon’s index (b), principal coordinate analysis (PCoA) plot based on Jensen-Shannon analysis (c), and phylum level (d).

(B) Effects on gut microbiota composition indicated by Cladogram between the feces of HVs and UC patients (a) and between the feces of HVs and CD patients (b). Data values were indicated as mean ± SD (HV-F n=6; UC n=9; CD n=6). Means with same letters are not significantly different (p < 0.05). (A)(a, b), Kruskal-Wallis test (nonparametric test).

**
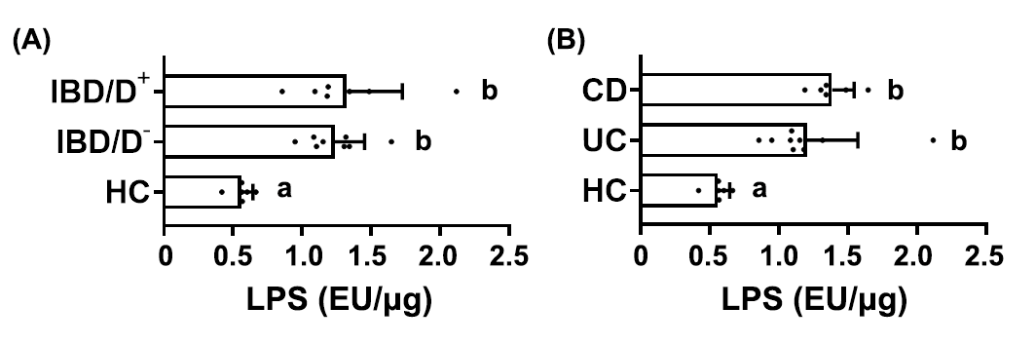
**

**Figure S5.** The LPS levels of HV-F and IBD-F. (A) LPS levels of HV, IBD/D^−^-F and IBD/D^+^-F. (B) LPS levels of HV, UC-F and CD-F. Data values were indicated as mean ± SD (HV-F n=6; UC n=9; CD n=6). Means with same letters are not significantly different (p < 0.05). (A) and (B), Kruskal-Wallis test (nonparametric test).

~~
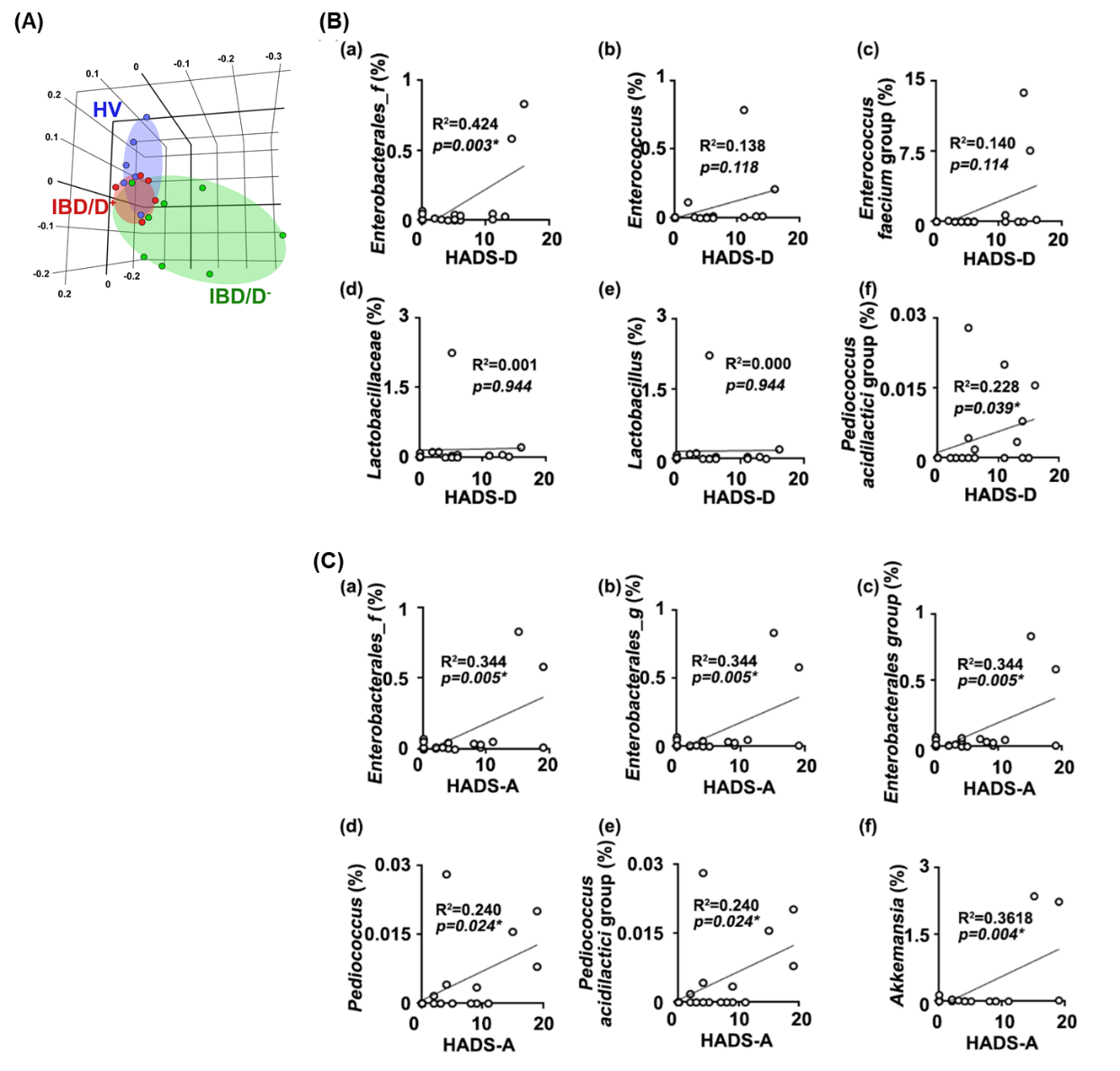
~~

**Figure S6.** The relationship between gut microbiota composition and HADS. (A) Effects on principal coordinate analysis (PCoA) plot based on Jensen-Shannon analysis. Two IBD/D+ patients thought to be outliers were excluded. (B) The relationship between HADS-D and gut microbiota composition (exclude two outliers): (a) Enterobacterales_f, (b) *Enterococcus*, (c) *Enterococcus faecium* group, (d) *Lactobacillaceae,* (e) Lactobacillus, and (f) *Pediococcus acidilactici* group. (C) The relationship between HADS-A and gut microbiota composition(exclude two outliers): (a) Enterobacterales_f, (b) Enterobacterales_g, (c) Enerobacterales group, (d) Pediococcus, (e) *Pediococcus acidilactici* group, and (f) Akkemansia. HV n=6; IBD/D^−^ n=8; IBD/D^+^ n=5.


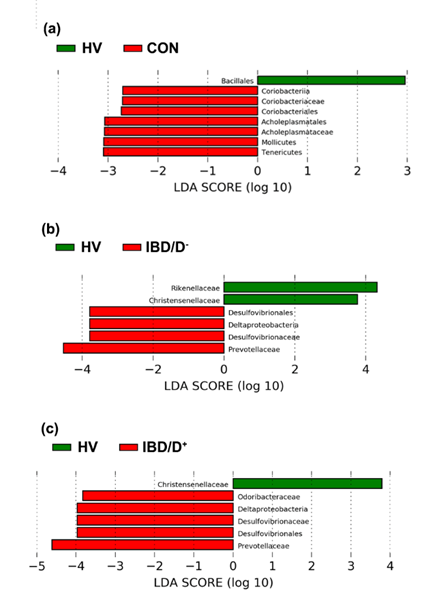


**Figure S7.** The fecal microbiota composition of mice orally transplanted with patients with IBD/D^−^ or IBD/D^+^ and healthy volunteers (HVs). Effects on the linear discriminant analysis (LDA) between HV and patients with IBD/D^−^ or IBD/D^+^. Data values were indicated as mean ± SD (NC n=6; HV-F n=4; IBD/D^−^ n=8; IBD/D^+^ n=7: each n value is the average obtained from 3 mice).


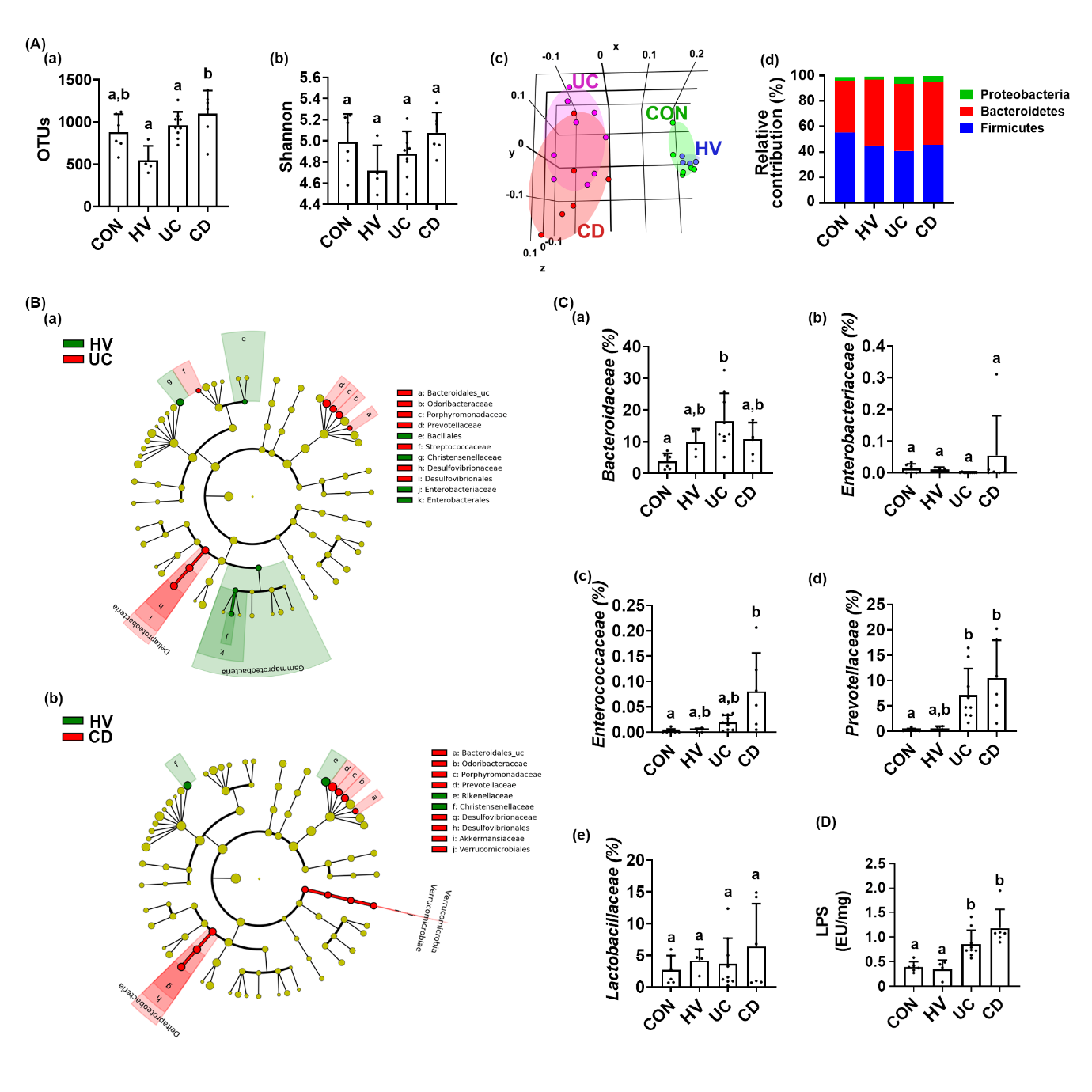


**Figure S8.** The fecal microbiota composition of mice orally transplanted with patients with UC, CD, or healthy volunteers (HVs). (A) Effects on OTU richness (a), Shannon’s index (b), principal coordinate analysis (PCoA) plot based on Jensen-Shannon analysis (c), and phylum level (d). (B) Effects on gut microbiota composition indicated by Cladogram between the feces of HVs and UC patients (a) and between the feces of HVs and CD patients (b). (C) Effects on the levels of families *Bacteroidaceae* (a), *Enterobacteriaceae* (b), *Enterococcaceae* (c), *Prevotellaceae* (d), and *Lactobacillaceae* (e). (D) Effects on the fecal LPS levels. Data values were indicated as mean ± SD ((NC n=6; HV-F n=4; UC n=9; CD n=6: each n value is the average obtained from 3 mice). Means with same letters are not significantly different (p < 0.05). (A)(a,b), (C)(a,b,d,e), and (E), Kruskal-Wallis test (nonparametric test); (B)(c), ANOVA with Tukey’s multiple comparisons test; (D)(a, b), One-way ANOVA Bonferroni’s multiple comparisons test (parametric test).








**Figure S9**. Fecal microbiota transplantation (FMT) from healthy volunteers (HVs) alleviated IBD/D^+^-F-induced depression and colitis in the transplanted mice. (A) Effects in IBD/D^+^-F (UC/D^+^-F- or CD/D^+^-F)-gavaged mice. (a) Effect on the occurrence of anxiety/depression in the marble burying task. (b) Effects on the IL-6 expression in the blood. Effects on the colon length (c), macroscopic score (d), stenosis score (e), and IL-1β (f) and IL-6 expression (g) in the colon. (h) Effects on the fecal LPS levels. (B) Effects in IBD/D^−^-F (CD/D^−^)-gavaged mice. Effects on the occurrence of anxiety/depression in the LDT (a) and MB tasks (b) and FST (c). Effects on the colon length (d), macroscopic score (e), and stenosis score (f) in the colon. HF, IBD/D^+^-F, or IBD/D^−^-F suspension was orally transplanted in mice once a day for 5 days. Control mice (CON) were treated with vehicle (saline) instead of fecal suspension. From the next day, HV-F suspension was gavaged in IBD/D^+^-HV and IBD/D^−^-HV mice once a day for 5 days. Con, IBD/D^−^, and IBD/D^+^ mice were treated with vehicle (saline) instead of fecal suspension. Data values were indicated as mean ± SD (n=6). Fecal Enterococcus sp. population was analyzed by using qPCR. Means with same letters are not significantly different (p < 0.05). All data were analyzed using ANOVA with Tukey’s multiple comparisons test.
